# The Effects of Anticipation and Visual and Sensory Performance on Concussion Risk in Sport: A Review

**DOI:** 10.1186/s40798-020-00283-6

**Published:** 2020-11-16

**Authors:** Stacey M. Kung, Titus K. Suksreephaisan, Blake G. Perry, Barry R. Palmer, Rachel A. Page

**Affiliations:** 1 School of Sport, Exercise & Nutrition, Massey University, Wellington, New Zealand; 2 School of Health Sciences, Massey University, Wellington, New Zealand

**Keywords:** Vision, Mild traumatic brain injury, Athletes, Training

## Abstract

Sports-related concussions pose a significant public health concern, and preventative measures are needed to help reduce risk in sport. Vision training could be a suitable prevention strategy for sports-related concussion to help improve athletes’ abilities to scan the visual field for oncoming objects or opponents and thus anticipate head impacts. By accurately anticipating impacts, athletes can prepare for impact or attempt to avoid the collision altogether. The purpose of this review is to explore the relationships between anticipation, visual and sensorimotor performance and head accelerations, as well as to examine the efficacy of vision training programmes in reducing concussion risk in sport. Anticipation of head impacts has been shown to help reduce linear and rotational head accelerations, particularly for mild-to-moderate severity head impacts, but less so for severe head impacts. There is conflicting evidence regarding the influences visual and sensorimotor performance and oculomotor behaviour have on concussion risk. However, preliminary research indicates vision training may help reduce concussion rates in collegiate American Football players. Therefore, this promising area of research warrants further investigation, particularly the role of anticipation and visual and sensory performance on reducing concussion risk in non-helmeted contact sports.

## Key Points

- Sports-related concussions pose a major public health concern internationally.
- Anticipation and visual performance may influence the severity and frequency of head impacts.
- There are potential benefits of vision training to aid in reducing concussion risk.
- Further research is needed to assess the role of anticipation and visual and sensory performance on reducing concussion risk, particularly in non-helmeted contact sports.

## Introduction

Concussions are a subset of traumatic brain injuries, which are induced by biomechanical forces, typically resulting in short-term neurological impairments. Following a concussion-inducing head impact, individuals may present with one or more symptoms such as headache, amnesia, irritability, slowed reaction times, sleep/wake disturbances, balance impairment, light sensitivity, tinnitus or loss of consciousness [1]. The combination of signs and symptoms vary between individuals and often dissipate in a matter of days or weeks, but can persist longer. With recurring concussions, individuals are susceptible to ongoing mild cognitive impairment, depression, behavioural changes or structural damage to the brain. Repetitive head impacts, regardless of whether they are concussive or sub-concussive hits, may result in chronic traumatic encephalopathy. More rarely, second-impact syndrome could result if initial symptoms do not resolve before a subsequent concussion occurs [2–4]. Sport and recreational activities are a major cause of concussions and present a serious public health concern worldwide [2, 5, 6]. In New Zealand, 16,994 sports-related concussions (SRCs) were reported in 2018–2019, comprising ~ 48% of all reported concussions and traumatic brain injuries during that period [7]. In the USA, it has been estimated that approximately 1.6–3.8 million SRCs occur annually [8]. In Canada, there were ~ 46,000 diagnosed concussions for 5–19-year-olds in 2016–2017; of these ~ 50% were sports-related [9].

The incidence of concussion is notably greater in collision and contact sports. Over a 10-year period in New Zealand, 1330 of 20,902 reported concussions from seven team sports were of moderate-to-severe intensity, which predominantly occurred in rugby union (60.3%), followed by soccer (13.8%), rugby league (13.5%), netball (5.6%), hockey (2.6%), touch rugby (2.6%) and softball/baseball (1.7%) [10]. Among American high school athletes, American Football had the highest concussion rates, with 9.21 concussions per 10,000 athletic exposures (AE) (i.e. one athlete participating in one practice or competition), followed by boys’ lacrosse (6.65 per 10,000 AEs) and girls’ soccer (6.11 per 10,000 AEs) [2]. Kontos et al. [11] reported a rate of 1.58 concussions per 1000 AEs in American youth ice hockey players. Rugby union and rugby league also have high concussion rates internationally among youth athletes, with reported concussion rates ranging between 0.2 and 14.7 concussions per 1000 player-hours [12]. Of the participants surveyed in the respective studies, 48–62% of adolescent or under 20 rugby union players [13, 14], 45% of elite rugby union players [15] and 57% of adolescents involved in the Gaelic Games [16] reported having sustained at least one SRC. Meanwhile, approximately one in five American adolescents involved in various competitive sports has reported sustaining at least one diagnosed concussion during their lifetime [17].

The incidence of SRC is continuing to increase, with reported statistics likely to underestimate the true incidence of concussion [18]. Various factors may contribute to the increasing incidence of SRC, including faster and stronger athletes [19] producing more severe impacts, or improved methods and means of recognising, diagnosing and reporting concussions. However, it is estimated that at least 20% of concussions in sport are not diagnosed or reported, especially because medical treatment is rarely sought following mild concussions [8, 20]. A misconception presumably contributing to the number of undiagnosed concussions is that concussions only occur when there is a loss of consciousness, but this only occurs 8–19% of the time [8, 13]. Indeed, athletes fail to report concussion symptoms to avoid mandatory stand down periods [15, 21]. Additionally, evidence-based clinical guidelines for diagnosing and managing paediatric concussions were only formed in 2018 [5]. Therefore, numerous paediatric SRCs may have remained undiagnosed or been mismanaged due to lack of definitive guidelines [13] and/or expertise [3] to adequately identify such injuries. Anonymous surveys also revealed that players commonly return to play before gaining medical clearance [13] or ignore advice to never return to play due to their concussion history [22]. Failing to report symptoms, diagnose concussions, appropriately manage concussion symptoms and/or gain medical clearance to return to play can result in recurring concussions. Alongside concussion education in sport and applying more stringent management plans for players with concussion, more work is needed to help prevent SRC.

Schneider et al. [23] highlighted a paucity of prospective studies investigating the efficacy of various equipment-, training- or education-related prevention strategies to reduce concussion risk. One area of recent interest is the use of vision training to improve an athlete’s ability to anticipate a collision and thus brace for impact to reduce the magnitude of head accelerations. This paper will outline common mechanisms of concussion injuries before reviewing the effects of anticipation and visual and sensory performance on concussion risk in sport.

### Mechanisms of Injury

Concussions are caused by either a direct impact to the head, face or neck or an impact to the body that transmits impulsive forces to the head [1]. Concussion-inducing head impacts more commonly occurs during competition than during training [2, 11, 24], presumably due to a higher intensity of game play. The most common mechanism of injury for SRC is player-to-player contact [2], particularly direct hits to the side of the head in American Football, soccer, ice hockey and Australian Football [25, 26]. While head-to-head impacts are frequent in helmeted sports and soccer, concussions sustained during rugby union, rugby league and Australian Football were most frequently caused by contact between the head and an opponent’s upper limb or upper body [27]. Differences in the mechanisms of injury are presumably due to sport-specific demands.

Direct contact or inertial forces experienced upon impact can result in excessive linear and/or rotational accelerations of the head [28]. Linear accelerations of the head have been correlated to peak pressures within brain tissue [29]. However, research has identified that rotational head acceleration plays a greater role in causing brain injuries and inducing damage to the brain tissue due to shear forces [28, 30]. Research has attempted to identify injury thresholds via modelling concussion-inducing head impacts; a linear head acceleration of 85 g and rotational head acceleration of 6000 rad · s^−2^ have been proposed as such injury thresholds [31]. These head accelerations are comparable to values measured in vivo for high school and collegiate level athletes [32, 33]. However, concussions have occurred at rotational head accelerations lower than these proposed injury thresholds, including rotational accelerations as low as 163.35 rad · s^−2^ [34]. Furthermore, American Football players have exhibited degenerative changes to the white-matter of their brain after repetitive head impacts that were below the proposed injury thresholds, when compared to healthy non-athlete controls [35]. The severity of concussion symptoms is also largely independent of the magnitude and location of the head impact [34]. As such, determining definitive injury thresholds and predicting when symptoms will resolve are not feasible. Therefore, it must be stressed how important preventative strategies are for reducing the severity and frequency of head impacts in sport.

Helmets and mouthguards are typically used for protection in collision sports, such as American Football and ice hockey. Although this protective equipment helps reduce the risk of superficial head injuries, skull fractures and dental damage, they have not decreased the incidence of concussions [23, 36]. In fact, using head gear appears to encourage more reckless or aggressive behaviour due to a false sense of protection [23, 37]. Helmets have also been found to inhibit peripheral reaction times [38], which could be a contributing factor to concussion risk, particularly because impacts to the side of the head are a common mechanism of concussion injuries. Neck stiffness and contraction latency of the neck musculature have been identified as modifiable factors, which provide dynamic stabilisation of the head and neck and thus reduce concussion risk [39, 40]. In order to elicit these protective responses, athletes need to be able to anticipate collisions during training and game play. Good vision and sensorimotor skills are thus necessary to either avoid impacts or prepare the body to reduce the severity of head impacts and head kinematics.

To further highlight the influence of vision on concussion risk, Clark et al. [41] observed poor eye discipline in female soccer players heading a ball compared to male players. Specifically, female soccer players were more likely to close their eyes while heading a ball than males. Heading the ball with closed eyes could increase concussion risk as there may be less awareness of the ball’s velocity, which would affect their ability to anticipate the head impact intensity and elicit an appropriate response to stabilise the head. While this study only assessed Google images to investigate sex differences in eye discipline [41], it indicates poor vision may be an important risk factor for concussions. Previous research has demonstrated the potential benefits of sports vision training to enhance a variety of sensorimotor performance outcomes [42] and sports performance [43, 44]. Vision training may thus be a useful tool to help improve eye discipline, gaze behaviour, and visual and sensorimotor performance to improve anticipation for collision avoidance and reduce concussion risk.

Anticipation and the ability to quickly process visual cues and elicit appropriate reactions are therefore hypothesised to help reduce the risk of direct head impacts. There are various components of anticipation, including pattern recognition, prioritisation of sensory cues, assignment of situational and event probabilities, gaze behaviour and peripheral vision [45]. Depending on the task and situation, the weighting of these peripheral-cognitive skills involved in anticipation differ [46]. Before assessing the efficacy of vision training programmes in reducing concussion risk, the relationships between anticipation, visual and sensorimotor performance and head accelerations need to be established. Therefore, the remainder of this paper will proceed to review the following: (1) the effects of anticipation on linear and rotational head accelerations, (2) whether visual and sensory performance influences concussion risk, and (3) if visual and sensory training can be used to effectively reduce SRCs. The review uses journal articles identified up to December 2019 through the PubMed, SPORTDiscus, MEDLINE, Web of Science and CINAHL Complete databases using combinations of the following search terms: ‘head acceleration’, ‘visual performance’, ‘visual training’, ‘vision training’, ‘anticipation’, ‘concussion’, ‘traumatic brain injury’, ‘sport*’ and ‘athlete*’. Additional papers were identified from reference lists of the articles found through the electronic searches. Only articles involving healthy participants were included to review the literature regarding the prevention of primary concussions.

## Effect of Anticipation on Head Acceleration

Numerous studies have assessed head accelerations and the activation of the neck musculature using a range of head perturbation methodologies including load dropping, quick release of head support and direct head impacts [40]. Load dropping and quick release methods rely more heavily on proprioceptive and vestibular feedback to elicit a reaction to perturbations, thus removing the use of visual cues to anticipate impacts. Therefore, the present review focuses on direct impact studies, which enable participants to use visual cues to anticipate a head impact and prepare for contact. Seven studies were identified that investigated the effects of anticipating a direct impact on head accelerations (Table 1). Two studies calculated head accelerations using computational modelling [51, 52], while five studies were assessed in vivo [30, 47–50]. In the in vivo studies, one methodology was strictly laboratory-based, two adopted sport-specific protocols involving typical training drills and two analysed head accelerations of concussion-inducing impacts during game play.

**Table 1:** Studies investigating the effects of anticipation on linear (g) and rotational (rad · s^−2^) head accelerations

	Participants, sport and level	Protocol	Anticipation condition	Linear head acceleration (g)	Rotational head acceleration (rad · s^−2^)	Muscle activity (mV)	Significant findings	Limitations
In vivo studies								
Kuramochi et al. [47]	9 healthy males 19–30 years	Direct impact to the forehead while sitting in a chair Impact: 4 kg weight released from 25° Uniaxial accelerometer Rectified EMG averaged over onset duration for SCM and TRP	Anticipated (eyes opened) Unanticipated (eyes closed)	3.85 ± 0.33 (37.8 ± 3.3 m · s^−2^) 3.72 ± 0.24 (36.5 ± 2.4 m · s^−2^)	N/A N/A	R/L SCM: 6.3 ± 1.5*/ 8.3 ± 2.5* R/L SCM: 17.9 ± 3.7/ 22.4 ± 5.4	Anticipation did not affect the linear head acceleration No difference in the onset latency between conditions Unanticipated condition elicited greater muscle activity than the anticipated condition	A strictly laboratory-based study; findings limited for extrapolating the results to concussive head impact intensities during game play Head accelerations significantly below the proposed 85 g injury threshold Limited to sagittal plane movement
Mihalik et al. [30]	16 male Bantam-level ice hockey players (elite youth) Average age: 14.0 ± 05 years Experience: 7.8 ± 1.7 years	Data collected from single team over 54 games: HIT system (> 10 g) Qualitatively assessed body position from video analysis (CHECC List) Location on the ice (along playing boards, or on the open ice)	Anticipated (‘saw hit coming’), good body position Anticipated, bad body position Unanticipated regardless of body position	20.7 21.4 22.6	1409.4* 1420.4* 1550.0	N/A N/A N/A	Anticipation was not associated with lower linear head accelerations For the medium intensity impacts, anticipation was associated with a reduced severity of rotational head acceleration Anticipation had no effect on head acceleration for the highest intensity collisions (> 75 percentile of collisions according to the HIT severity profile)	Small convenience sample: various player positions, single team, 1 season Investigator’s judgement on the player’s anticipation status from video footage only
Hasegawa et al. [48]	12 male high school rugby players Average age: 16.8 years	5 rugby tackles to the chest Triaxial accelerometer measured head accelerations of the attacker and defender EMG to assess bilateral masseter and SCM^1^	No clenching instruction (unanticipated) Tightly clenched (anticipated)	A: 2.64 ± 0.33 D: 2.86 ± 0.23 A: 2.16 ± 0.50* D: 2.30 ± 0.27*	N/A N/A	Masseter A/D: 0.22 ± 0.12/0.29 ± 0.18 SCM A/D: 0.60 ± 0.19/0.70 ± 0.23 Masseter A*/D*: 0.55 ± 0.25/0.73 ± 0.46 SCM A/D: 0.62 ± 0.18/0.77 ± 0.24	Clenching increased activity of the masseter muscle and decreased linear head acceleration Muscle activity onset occurs prior to body contact	Small sample size Analysed sub-concussive intensity head impacts to minimise injury risk; cannot confidently extrapolate results to concussive head impact intensities Rotational head accelerations not analysed
Narimatsu et al. [49]	11 male high school soccer players Average age: 17.2 years	5 trials of heading a soccer ball under 3 conditions Triaxial accelerometer measured head accelerations EMG ^1^ to assess bilateral masseter and SCM Ball projected approx. 9 m from JUGS soccer machine (initial motor velocities set at 28 m · s^−1^ (RM) and 38 m · s^−1^ (LM)	No clenching instruction (unanticipated) Clenched w/o mouthguard (anticipated) Clenched with mouthguard (anticipated)	28.4 ± 7.0 23.9 ± 6.2* 21.5 ± 4.6*	N/A N/A N/A	Masseter: 44.0 ± 38.2 SCM: 68.6 ± 47.7 Masseter: 132.7 ± 76.5* SCM: 133.5 ± 74.2* Masseter: 154.0 ± 99.3* SCM: 159.3 ± 76.8*	Clenching in anticipation of heading a soccer ball increases muscle activity of masseter and SCM muscle compared to the no clenching instruction condition only (no mouthguard and clenching interaction), and reduces linear head acceleration Masseter and SCM active before headed ball	Small sample size Not game-like intensity, intentionally eliciting lower intensity head impacts to minimise head injury risk; cannot confidently extrapolate to concussive head impact intensities
Schmidt et al. [50]	32 male high school conference 3A varsity American Football players Average age: 16.7 ± 0.9 years	Data collected during game play HIT system to assess head accelerations Video analysis to subjectively classify impacts as anticipated or unanticipated	Anticipated Unanticipated	25.9 26.5	1605.5 1621.3	N/A N/A	There was a trend (*p* = 0.07) towards a lower linear head acceleration when the impact was perceived to be anticipated	Small convenience sample: various player positions, single team, over 1 season Limitations of video analysis for determining anticipation level No assessment of concussion risk No assessment of whether perceived anticipation resulted in players moving into a protective position
Simulations and modelling								
Jin et al. [51]	Simulated impacts using finite element model Head and neck complex of the Global Human Body Model with validated helmet model 27 pairs of Hill-type muscle elements	Reconstructed concussive and non-concussive American Football-related head impacts Magnitude and timing of impact for simulation and experimental data within 10% of each other; peak impact force approx. 10,000 N 4 conditions simulated	(1) No muscle activity (2) Reactive muscle response (onset at impact, 55 ms to peak activation) (3) Pre-activation response (40 ms before impact) (4) Pre-activation response (40 ms before impact) with 200% strength	113.36 112.18 111.75 110.59	29.25 rad · s^−1^ 26.90 rad · s^−1^ 22.72 rad · s^−1^ 20.16 rad · s^−1^	N/A N/A N/A N/A	Anticipatory activation of neck musculature reduced injury criteria No change in the injury criteria with double muscle strength, or reactive activation compared to no activation No differences in linear accelerations of the head Pre-activation reduced peak rotational velocity (18.1–31.0%)	Theoretical computational model only Results largely influenced by the model constraints used
Eckersley et al. [52]	Simulated model Duke University Head and Neck model	Simulated 4 head impact conditions with 6 neck muscle activity conditions at 8 impact sites Impacts: (1) Impact of high speed object in flight (2) 80 g helmet-to-helmet impact (short duration) (3) 80 g helmet-to-helmet impact (longer duration) (4) 40 g helmet-to-helmet impact	(1) Relaxed (min. activation to maintain head stability) (2) Maximally activated neck musculature (3) Maximally activated neck flexors (4) Maximally activated neck extensors	N/A N/A N/A N/A	Lowest value ↑ ↑ ↑	N/A N/A N/A N/A	Magnitude of cervical muscle force does not influence short-term (< 50 ms) head kinematics Impacts to the side of the head, higher than ear level consistently produced highest peak resultant angular acceleration Musculature presumably active at time of impact to simulate pre-activation Values not reported	Theoretical computational model only Results largely influenced by the model constraints used Estimated constraints used to simulate a direct impact to the head, rather than using data from real concussive head impacts

*A* attacker, *D* defender, *HIT* head impact telemetry, *LM* left motor, *RM* right motor, *SCM* sternocleidomastoid muscle activity, *TRP* trapezius

^1^Details of the EMG analysis not provided

*Significant difference compared to the unanticipated condition

The studies that simulated concussion-inducing head impacts through computational modelling produced conflicting results. Jin et al. [51] simulated a head impact to the side of the head, which was informed by concussion-inducing head impact data previously observed in American Football. The study compared four conditions to determine the effects of reactive and anticipatory pre-activation of the neck musculature, as well as the effect of neck strength to help reduce head accelerations. From these simulations, no differences in peak linear head accelerations were observed across conditions. However, there was a reduction in the head’s angular velocity with reactive activation of the neck musculature (i.e. onset of activation occurred upon impact), compared to no muscle activation. There was an even greater reduction in the head’s angular velocity with a pre-activation response (i.e. muscle onset 40 ms prior to head impact) compared to no muscle activity. These results suggest that bracing the head and neck in anticipation of an impact would help improve dynamic stabilisation of the head, which could help reduce concussion risk. In contrast to these results, Eckersley et al. [52] failed to demonstrate a protective anticipatory effect on head accelerations. Specifically, this comprehensive study assessed 192 simulations including four head impact conditions across eight impact sites and four activation conditions and compared three head and neck models. Their results indicated that anticipatory muscle activity did not reduce rotational head accelerations [52]. Surprisingly, the simulations with maximally activated neck musculature elicited greater head accelerations than the ‘relaxed’ condition, which had the minimal level of activation necessary to support the head. However, there was no comparison of reactive and pre-activation responses in the latter study, only a comparison of maximally and minimally activated neck musculature. However, individuals would be unlikely to exhibit no dynamic muscle response following an impact. Differences between these two studies may partly be explained by differences in the head and neck models used for the simulations and the model constraints used [51, 52]. Eckersley et al. [52] argued that the model used by Jin et al. was too simplistic and would result in an unstable positioning of the head. However, rather than deriving actual head impact data, such as time history of linear and rotational head accelerations for the simulation [51], 40-g and 80-g impacts were used by Eckersley et al. [52] to represent mild and moderate head impacts, respectively. Further research is needed to quantify a wider range of head impacts and the effects of pre-activation (i.e. anticipation) and reactive (i.e. unanticipated) muscle activation.

The laboratory-based study conducted by Kuramochi et al. [47] assessed a direct impact to the forehead using a weighted ball. To simulate anticipated and unanticipated conditions, participants either had their eyes opened or closed, respectively. Peak linear head acceleration and muscle activity of the sternocleidomastoid were analysed using electromyography (EMG). No differences in head accelerations were observed between anticipated and unanticipated conditions. Interestingly, there were no differences in onset latency times of the sternocleidomastoid following the head perturbation, but the level of muscle activity was higher in the unanticipated condition. As there was a consistent onset latency time, it is assumed the participants did not brace for impact prior to the head impact, even when the participants could see the ball approaching. It is unclear why there was no bracing behaviour observed, particularly for the anticipated condition. As participants were familiarised with the protocol, they may have perceived the head impact severity did not warrant pre-activation of the neck musculature. However, for safety purposes, linear head accelerations elicited in their study were well below the proposed injury thresholds (i.e. less than 4 g versus 85 g respectively). Due to the strictly laboratory-based nature of this study and low head accelerations elicited, extrapolation of these findings to sport-specific applications is limited.

For greater sport specificity, head accelerations have also been assessed during rugby union tackling drills [48] and heading a soccer ball [49]. These studies used teeth clenching as a form of anticipation because it elicits greater activation of the masseter muscles, which is thought to improve neck and head stability [49]. In both studies, linear head accelerations decreased with teeth clenching, coinciding with greater activation of the masseter muscles, but no change in sternocleidomastoid activity [48, 49]. When comparing clenching with and without a mouthguard, greater EMG activity of the masseter muscles was observed with a mouthguard along with a further reduction in linear head acceleration while heading a ball [49]. However, reductions in linear head accelerations with greater activation of the masseter muscles were only seen at head accelerations below the 85-g concussion-inducing impact threshold (i.e. < 3 g [48] and < 30 g [49]). From these studies, it is unclear whether clenching would provide sufficient head stability during more severe head impacts. These studies also failed to report on rotational head accelerations, which have been argued to be a more important risk factor for concussions [28, 30].

Head accelerations of actual concussion-inducing head impacts during game play were assessed in youth ice hockey [30] and high school American Football [50] players. In these studies, head impacts were assessed using the Head Impact Telemetry (HIT) system, with the units placed inside athletes’ helmets. Both studies also used video analysis to subjectively determine if players anticipated the impact. Anticipation did not affect linear head accelerations for the ice hockey or American Football players. Among the youth ice hockey players, anticipation of medium intensity head impacts reduced the rotational head accelerations compared to unanticipated head impacts [30]. However, there were no protective effects of anticipation on rotational head accelerations for severe head impacts. There was also a lack of difference in the rotational head accelerations between anticipated and unanticipated impacts in high school American Football players [50]. On average, the rotational head accelerations recorded for the American Football players (1605–1621 rad · s^−2^ [50]) were greater than those observed in youth ice hockey players (1409–1550 rad · s^−2^ [30]). As such, the protective effects of bracing for impact may also be limited for severe impacts in American Football. Additionally, the qualitative assessment of player anticipation did not assess whether the player managed to position themselves in a manner that protected their head and neck when the collision was anticipated [50]. This information would help determine if a bracing or protective reaction in response to anticipating a collision reduces head impact severity.

## Effects of Visual and Sensorimotor Performance on Concussion Risk

Vision is the dominant sensory system and plays an important role in avoiding collisions [45, 53–55]. Specifically, vision is needed to recognise potential oncoming dangers and requires accurate tracking and prioritisation of relevant visual cues. Compared to novices, experienced athletes often fixate on a greater number of locations for shorter durations [46]. The use of peripheral vision also enables individuals to process multiple visual cues at the same time without having to shift the gaze position [56]. Differences in gaze behaviours suggest more exploratory and analytical behaviour is used by more experienced players when scanning the visual field. Less-experienced players may be more likely to miss relevant cues, making them more susceptible to sustaining a collision or head impact.

Hindered peripheral reaction time with helmet use [38] and poor eye discipline [41] appear to increase the risk of sustaining a concussion. Without being able to anticipate body contact, individuals are unable to avoid collisions, position themselves in a protective stance or brace for impact, thus increasing the risk of injury. As such, visual performance and gaze behaviours would presumably play an important role in avoiding contact or reducing head impact severity. However, few studies have investigated whether visual and sensorimotor performance and oculomotor behaviour affects concussion risk in sport (Table 2).

**Table 2:** Studies investigating the effects of visual and sensorimotor performance on concussion risks

	Participants	Assessments	Relationships between visual and sensory performance and head impacts					Findings	Limitations
			Visual and sensory measure	Linear head acceleration	Rotational head acceleration	HIT severity profile	Head impact frequency		
Harpham et al. [57]	38 Div I college American Football players (20.4 ± 1.4 years; 190.2 ± 6.7 cm; 109.3 ± 17.8 kg)	Nike SPARQ sensory station Head Impact Telemetry system General linear mixed models to test relationship between visual performance and impact severity	Visual clarity (SVA) Contrast sensitivity Depth perception Near-far quickness Target capture (DVA) Perception span Eye-hand coordination Go/No go decision making Reaction time	– ↓ Risk ↓ Risk ↓ Risk – – ↓ Risk ↓ Risk	– – – ↓ Risk ↓ Risk ↓ Risk ↓ Risk ↓ Risk ↓ Risk	N/A	N/A	High performers on certain assessments were at lower risk of concussion	Small convenience sample: various player positions, single team, 1 playing season Arbitrary cut-offs for ‘high’ and ‘low’ performers on Nike SPARQ station
Schmidt et al. [58]	37 male high school American Football players (16.59 ± 0.89 years; 180.35 ± 6.39 cm; 87.18 ± 19.03 kg)	Nike SPARQ sensory station Head Impact Telemetry Assessed odd ratios for sustaining moderate and severe head impacts	Visual clarity (SVA) Contrast sensitivity Depth perception Near-far quickness Target capture (DVA) Perception span Eye-hand coordination Go/No go decision making Reaction time	– – – – – – – – ↑ Odds^2^	– – – – – – – – ↑ Odds^2^	– – – ↑ Odds^1^ – – – – ↑ Odds^1^	N/A	Using a median split to classify high and low performers, higher performers did not reduce the odds of sustaining high-magnitude impacts	Small convenience sample: various player positions, single team, 1 playing season Arbitrary cut-offs for ‘high’ and ‘low’ performers on Nike SPARQ station
Kiefer et al. [53]	12 male high school ice hockey players (16.50 ± 1.17 years; 177.79 ± 6.83 cm; 70.32 ± 7.19 kg)	Oculomotor performance Head acceleration 3 tasks: - Prosaccade task - Self-paced saccade task - Smooth pursuit task	Prosaccade latency Prosaccade latency variability Self-paced saccade velocity Self-paced saccade initial error Medium-speed smooth pursuit latency Medium-speed smooth pursuit gaze velocity variability Fast-speed smooth pursuit gaze velocity variability	N/A	N/A	N/A	– ↓ Risk ↑ Risk – – ↓ Risk –	More variable oculomotor reaction time, faster saccadic eye motion and more variable gaze velocity when following a predictable target trajectory were related to an increased risk of head impacts Higher variability of saccade latency and smooth pursuit tracking may indicate a lack of attention to task-relevant visual cues necessary to avoid collisions There were no changes in concussion risk when accounting for accuracy of the self-paced saccade task	Small convenience sample: various player positions, single team, 1 playing season Combination of anticipated and unanticipated hits analysed; no analysis of whether the impact was anticipated or not

^1^For moderate (HITsp: 11.7–15.7) and severe (HITsp: ≥ 15.7) head impacts

^2^For moderate (HITsp: 11.7–15.7) head impacts, but only tended to increase the odds

*DVA* dynamic visual acuity

Schmidt et al. [58] and Harpham et al. [57] used similar methods to measure visual performance, as well as head impacts during practices and game play of high school and Division I college American Football players, respectively. Specifically, they assessed visual performance using the Nike SPARQ Sensory Station and used the HIT system to measure linear and rotational head accelerations. The Nike SPARQ sensory station is a visual and sensory training tool designed for athletes. The system is computer based and consists of an interactive touch screen device and two high-resolution LCD monitors that assess nine visual and sensorimotor domains. The visual sensitivity tests are Static Visual Acuity, Contrast Sensitivity and Depth Perception. The eye quickness tests are Near-Far Quickness and Dynamic Visual Acuity. The visual-motor control tests are Perception Span, Eye-Hand Coordination, Go/No-Go and Hand Response Time. Detailed methods for completing the aforementioned tests using the Nike SPARQ are given by Erickson et al. [59] and Wang et al. [60]. These tests generally have good inter-session reliability, but the Near-Far Quickness, Eye-Hand Coordination and Go/No-Go tests have been found to be influenced by learning effects [59]. The static visual acuity and contrast sensitivity tests have been cross-validated [60]. The validity of the other assessments is yet to be determined.

Better performance in the sensorimotor measures relating to eye quickness, visual-motor control and depth perception was associated with less severe head impacts among collegiate American Football players [57]. These factors require quick and accurate processing of central and peripheral cues. In the sporting context, performing better in these domains would help scan the visual field for relevant cues indicating there are oncoming players or objects. Players would subsequently have more time to produce appropriate motor responses to protect themselves. Conversely, better visual and sensorimotor performance did not reduce head impact severity among high school players [58]. In fact, high visual performers among high school athletes had greater odds of sustaining more severe head impacts if they performed better in the near-far quickness and hand response time tests. A number of reasons may contribute to the conflicting results between these two studies.

Within an American Football team, different playing positions require different skillsets and are exposed to different visual stimuli and playing situations. A lack of significant findings among high school players [58] could be due to the greater heterogeneity of American Football skills among the players, both within and between playing positions compared to Division I collegiate players. There were also uneven numbers of players within position groups (line positions: *n* = 14; skill positions: *n* = 23), who were also unevenly split between the high- and low-performing groups. There could be additional factors contributing to the severity of head impacts among high school players that were unaccounted for when assessing the influence of visual performance and reaction time alone. Younger players may be more susceptible to sustaining concussions due ongoing development of the nervous and musculoskeletal systems [61, 62]. Additionally, good sensorimotor skills under controlled conditions do not necessarily correspond to a better ability to avoid collisions or protect themselves during game play. For example, better reaction times assessed by the Nike SPARQ Sensory Station in high school players increased the odds of sustaining more severe collisions. If defensive players can react more quickly to the actions of the opponent, the defensive players could commit to, and initiate, contact with the offensive player sooner. While American Football players showed no differences in head accelerations between offensive and defensive collisions [50], this could differ in sports like rugby union where head collisions with opponents’ arms or upper body are more common [27] and be dependent upon proper tackling technique. The Nike SPARQ assessment of hand response time did not correlate with a clinical reaction time test, which involved catching an object using a pinch grip. While the clinical reaction time test has been associated with a functional head-protective response [63], the Nike SPARQ test has not been validated for such a protective response. The tested visual-motor control measures involved hand and arm responses, but not lower extremity or whole body movements. The latter movements may be just as important, if not more, to enable players to move out of the way of an oncoming opponent or object. Additional assessments of more task-specific reaction time tests may be needed to test the influence of sensorimotor performance on concussion risk.

Differences in visual and sensorimotor performance scores may have also contributed to the conflicting results, with respect to head impact severity among high school and collegiate players [57, 58]. Myelination in the sensorimotor system continues into adulthood, and depth perception does not mature until early adolescence, which could affect performance and spatial awareness [64]. It is possible that neither the high- nor low-performing high school players scored as well as the high-performing collegiate players whose visual performance was associated with a lower risk of sustaining severe head impacts. However, visual performance scores were not provided by Harpham et al. to make direct comparisons between studies. Inhibited peripheral reaction time associated with helmet use [38] may also affect high school players to a greater extent than collegiate players. However, differences in the effect helmets have on visual and sensorimotor performance between high school and collegiate players have not yet been investigated. Conversely, Division I collegiate players may be more successful at using any slight advantage in visual performance to improve anticipation of impacts than high school athletes. They may also have better pattern recognition with respect to oncoming dangers so more rapid protective reactions may be elicited. More research is needed to further investigate differences in risk reduction relating to visual and sensorimotor performance and to validate the use of Nike SPARQ Sensory Station tests with respect to predicting head impact severity and concussion risk.

In the studies of Schmidt et al. [58] and Harpham et al. [57] mentioned above, participants were somewhat arbitrarily divided into ‘high’ and ‘low’ performers (i.e. top 50% and bottom 50% of the players assessed, respectively). The lack of theoretical reasoning for the high- and low-performing groups is a clear limitation of these studies, and inclusion of performance indices may inform if players are at greater risk of injury. Identifying at-risk players would allow coaches to seek additional interventions to help address player-specific risk factors. Moreover, the relationship between visual and sensorimotor performance and anticipation requires further investigation to ensure there is a strong relationship between these factors. If these factors are not related, then interventions aimed at improving visual and sensorimotor performance would not help to improve anticipation.

Another aspect of anticipation is gaze behaviour and prioritisation of sensory cues. In order to determine the likelihood of an impact for collision avoidance, individuals need to quickly and accurately scan the visual field by rapidly switching visual focus between various opponents and objects. These brief and simultaneous movements of the eyes between fixations on various targets are called saccadic movements. Kiefer et al. [53] assessed oculomotor performance, including the speed and accuracy of eye movements, and their association with concussion risk in high school ice hockey players. The players completed three tasks, consisting of a prosaccade task, a self-paced saccade task and a smooth pursuit task. The prosaccade task involved following a central target as it moved left or right of the centre point as quickly and accurately as possible. The self-paced saccade test involved looking back and forth between two stationary targets on the screen as quickly and accurately as possible. The smooth pursuit task involved following the target’s sinusoidal trajectory that moved at three speeds. Seven oculomotor variables were chosen for analysis, and linear regressions between these variables and head impact frequencies from game play were performed (Table 2). Their results suggested that poor performance in three oculomotor outcome measures were associated with more frequent head impacts. Specifically, greater variability in both the prosaccade latency and smooth pursuit gaze velocity was suggested to indicate a lack of attention given to task-relevant visual cues. Consequently, a greater attentional load would be required to correct these highly variable oculomotor movements, thus reducing the ability to scan for a greater range of oncoming opponents and objects. Additionally, faster switching of fixation between targets during the self-paced saccade task increased the risk of sustaining more head impacts. Saccades interrupt the processing of visual information directly before, during and after a saccadic movement [56]. Eye movements that are too quick may thus hinder the ability to accurately process visual cues and detect approaching players. Gaze behaviours and oculomotor performance that allow more accurate scanning of the central and peripheral fields of vision could play an important role in successfully avoiding head impacts.

## Can Visual and Sensorimotor Training be Used to Effectively Reduce Concussion Risk?

The limited research available has provided conflicting results regarding the potential use of sensorimotor training to reduce concussion risk in sport. While anticipation was suggested to reduce rotational head accelerations in moderate intensity impacts in youth ice hockey players [30], this association was not as clear among high school American Football players, who also experienced higher impact severities [50]. High school American Football players with better visual and sensorimotor performance also failed to exhibit a reduced risk of SRC, compared to those who performed more poorly [58]. However, there was some evidence that supports the use of visual and sensorimotor training to reduce concussion risk in high school ice hockey [53] and Division I collegiate American Football players [57]. Specifically, lower risk of severe impacts was associated with better eye quickness and visual-motor control factors, as well as more efficient oculomotor performance. These studies [53, 57] provide initial support for using visual and sensorimotor training interventions to assist with reducing concussion risk. By improving visual awareness, pattern recognition and reaction time, athletes may be more prepared and better equipped with the skills necessary for successful collision avoidance or protecting themselves from impending impacts. However, these potentially protective effects may only be apparent among more experienced players [53, 57]. For less-skilled players, acquiring the skills necessary to brace for impact or improve their recognition of relevant visual cues may be necessary before starting visual training interventions.

Vision training has successfully improved visual and sensorimotor performance [42] and sport-specific performance [43, 44]. There are a range of programmes and drills that can be used for sports vision training. Clark et al. [65] describe a range of vision training methods using the Dynavision light board vision system, Brock’s String, the EYEPORT training system, accommodative flippers, tachistoscope, pinhole or strobe glasses, saccadic eye movement training, near-far training and stereopsis. Similar training methods were used for a pre-season conditioning programme with ongoing maintenance training during the baseball season, which improved batting performance among collegiate baseball players [43]. Preliminary results have also demonstrated that vision training involving the Dynavision light board and strobe glasses have the potential to improve functional peripheral vision and reduce the concussion rate among collegiate American Football players [66]. These drills were suggested to help improve the athlete’s awareness of the visual field and ability to see and respond to peripheral visual cues. However, the study had no control group for comparison. A controlled prospective study is required to confirm these results and to determine whether there is a causal relationship between vision training and concussion risk. Furthermore, studies also need to assess whether such programmes are effective at reducing concussion risk in other sports.

Vision training using the Nike SPARQ Sensory Station has also been used to effectively improve visual-motor function, but it was not as effective at improving visual sensitivity [67]. An 11-week vision training intervention involving Nike SPARQ Sensory Station drills with additional visual training drills also improved eye quickness and Go/No-Go performance in female collegiate softball athletes [42]. As visual-motor control and eye quickness factors were more relevant to reducing concussion risk [57], the Nike SPARQ Sensory Station could be used as a suitable vision-motor control training intervention. However, the efficacy of its use in reducing concussion risk in sport requires investigation.

When implementing visual training programmes, it is important to ensure that drills involve higher cognitive processing skills, along with the coupling of visual feedback and appropriate sport-specific motor responses. While the focus of this review has been on vision and anticipation, these factors in isolation cannot reduce concussion risk. Athletes also need to improve their ability to predict event likelihoods, anticipate collisions, make quick and appropriate decisions and subsequently produce an appropriate motor response for avoidance or to brace the head and neck [45, 55]. Adopting a more holistic approach may be most effective at reducing concussion risk. Thus, visual and sensorimotor training to improve anticipation along with improving neck strength and stiffness, reducing the onset latency of the neck musculature and training protective positioning for an impending collision may be a more effective approach to help prevent concussions in sport.

## Future Research

This research area is still in its infancy, with few studies investigating concussion risk at game-like intensities [30, 50] and laboratory-based studies limited to low head accelerations due to ethical reasons [47–49] (Tables 1 and 2). Further research is needed to confirm if these potentially protective effects of anticipation and better visual-motor skills exist across a wider range of sports and levels of competition involving larger and more homogenous samples. It would be particularly beneficial to conduct more studies that collect data from gameplay to gain a better understanding of the risk factors of concussions in various types of team sports, using similar study designs to those of Mihalik et al. [30] and Schmidt et al. [50] who measured head accelerations of concussion-inducing impacts. However, future studies may benefit from also obtaining player reports of anticipation, and body position where possible, rather than being limited to subjectively assessing these factors from video alone. As peripheral vision may be necessary to avoid head impacts to the side of the head, the influence of peripheral reaction time on head impact frequency and magnitude requires further investigation. The Nike SPARQ tests were limited to assessing peripheral vision on a 42-inch screen positioned in front of the participants. Tests that assess more extreme ranges of the peripheral field of vision may be beneficial for understanding the risk factors of lateral head impacts. Various sporting helmets have been reported to affect eye-hand coordination [68], while ice hockey helmets specifically reduce visual clarity and hand reaction time [68], and American Football helmets compromise vision and peripheral reaction times [38]. Yet, studies have focused on investigating sports involving helmet use (i.e. American Football [57, 58] and ice hockey [53]). To gain further information about the influence peripheral vision has on concussion risk, an area of interest is thus sports that do not involve the use of helmets and head gear to improve our understanding of the influence vision-related factors have on concussion risk in other high concussion risk sports (e.g. rugby union, soccer). It would be expected that players would exhibit a similar, if not exaggerated, advantage of better visual performance on concussion risk in non-helmeted sports where vision is not compromised. If that were the case, visual training may be more effective in reducing concussion risk among athletes in non-helmeted sports. As the mechanisms of injury are sport-specific, it also needs to be determined whether certain visual and sensorimotor training programmes are more effective than others depending on the sport in which they are implemented. In addition to vision training, coaches may want to integrate training on how to respond in situations where collisions are unavoidable. Although insignificant, there was a tendency for lower rotational head accelerations to be recorded when youth ice hockey players anticipated a collision and moved into a protective stance [30]. Players may benefit from learning how to position their body to protect their head and neck if they anticipate a hit and perceive a lack of time to avoid the collision. These skills would complement visual and sensorimotor training to reduce concussion risk, so that athletes can improve their ability to recognise visual cues indicating danger, anticipate a collision and quickly react to either avoid the collision or brace for impact.

## Conclusions

This review highlights a paucity of research investigating the relationships between visual and sensorimotor performance, anticipation and concussion risk. Studies have largely focused on sports where helmets are required. Although there are few studies available, there appears to be a potential effect of visual performance, oculomotor behaviour and anticipation on the frequency and severity of head impacts in helmeted sports, particularly among highly skilled players. Specifically, better visual-motor control and more effective oculomotor performance allow players to anticipate collisions, which appears to help mitigate the severity and frequency of head impacts. Sports vision training seems to be a promising method to improve visual-motor control and eye quickness that may assist with reducing concussion risk. Visual and sensorimotor performance thus warrants further exploration with regards to its effect on anticipation and mitigating concussion risk, particularly in sports where vision is not compromised by helmet use. The efficacy of visual and sensorimotor training programmes to reduce concussion risk across a wider range of sports and levels of competition also requires further investigation.

## Data Availability

Not applicable.

## Abbreviations

EMG: Electromyography
HIT: Head Impact Telemetry
rad · s^−2^: Radians per second squared
SPARQ: Speed, Power, Agility, Reaction and Quickness
SRC: Sport-related concussions
